# Clinical significance of lesion conspicuity on contrast-enhanced mammography

**DOI:** 10.1007/s00330-026-12660-y

**Published:** 2026-06-13

**Authors:** Tali Amir, Carol H. Lee, Molly P. Hogan, Sarah Eskreis-Winkler, Varadan Sevilimedu, Daniel J. Long, Noam Nissan, Victoria L. Mango, Kimberly N. Feigin, Maxine S. Jochelson, Christopher E. Comstock, Janice S. Sung

**Affiliations:** 1 Department of Radiology, Memorial Sloan Kettering Cancer Center, New York, NY USA; 2 Department of Epidemiology and Biostatistics, Memorial Sloan Kettering Cancer Center, New York, NY USA; 3 Department of Medical Physics, Memorial Sloan Kettering Cancer Center, New York, NY USA; 4 Department of Radiology, Sheba Medical Center, Tel Hashomer, Israel; 5 Breastlink Women’s Imaging; RADNET Beverly Hills, Los Angeles, CA USA; 6 Department of Radiology, New York-Presbyterian/Weill Cornell Medical Center, New York, NY USA; 7 Department of Radiology, Columbia University, New York, NY USA

**Keywords:** Contrast-enhanced mammography, Conspicuity, Breast malignancy, BI-RADS

## Abstract

**Objective:**

To determine if lesion conspicuity on contrast-enhanced mammography (CEM) is independently associated with malignancy.

**Materials and methods:**

This retrospective, single-institution study identified consecutive abnormal screening and diagnostic CEMs between January 2019 and December 2021. The conspicuity of enhancing lesions on CEM recombined images was graded (low, moderate, or high) by one or both breast radiologists assigned as readers for the study, blinded to two-year imaging follow-up or biopsy pathology results. The positive predictive value (PPV) for malignancy was determined for each level of conspicuity.

**Results:**

Across 476 CEM examinations in 455 women (median age, 49 years; IQR: 44–57), there were 563 enhancing lesions (55 malignant, 508 benign). Of the 563 enhancing lesions, 52% (292/563) were low conspicuity, 38% (213/563) moderate, and 10.3% (58/563) high. The PPV to predict cancer was 9% (95% CI: 6–12%), 11% (95% CI: 7–16%), and 10% (95% CI: 4–21%) for low, moderate, and high conspicuity (low vs high, *p* = 0.68; low vs moderate, *p* = 0.34; moderate vs high, *p* = 0.83). Of the 55 cancers, most high-conspicuity cancers presented on both recombined images and low-energy images (5/6; 83%), while most low- (21/25; 84%) and moderate-conspicuity cancers (17/24; 71%) presented on recombined images only (*p* = 0.006). Inter-reader agreement was almost perfect for 100 lesions graded by both breast radiologists (κ = 0.94).

**Conclusion:**

Lesion conspicuity is not independently associated with malignancy. Most enhancing lesions, including most cancers, show low or moderate conspicuity on CEM.

**Key Points:**

***Question*** *Is lesion conspicuity on CEM, determined on the recombined images, an independent predictor of malignancy*?

***Findings*** *The PPV for low, moderate, and high conspicuity to predict malignancy was 8.6%, 11.3%, and 10.3%, respectively (non-significant differences between low, moderate, and high conspicuity)*.

***Clinical relevance*** *Lesion conspicuity is not an independent predictor of malignancy. Enhancing lesions seen on recombined images should not be considered benign based on low conspicuity alone, as most low-conspicuity cancers presented with enhancement only and lacked a correlate finding on low-energy images*.

**Graphical Abstract:**

**Figure:**
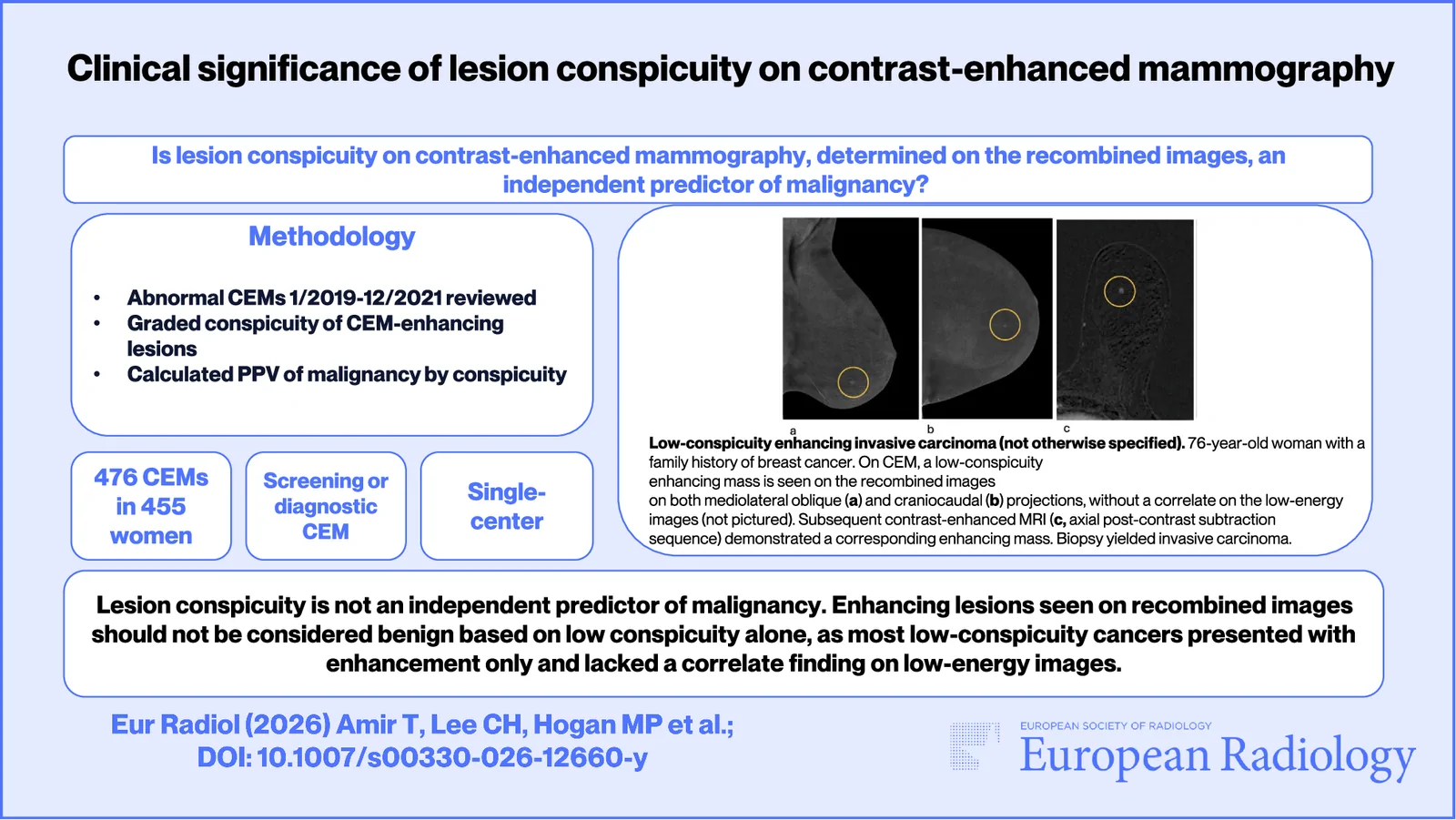

## Introduction

Contrast-enhanced mammography (CEM), a vascular-based breast imaging modality approved by the United States Food and Drug Administration (FDA) in 2011, was used initially as a diagnostic tool [1–5] but has since been used in breast cancer screening [6–10]. CEM encompasses both a low-energy image, equivalent to a full-field digital mammogram [11], and a recombined image that highlights areas of contrast enhancement, similar to MRI. The American College of Radiology breast imaging reporting and data system (BI-RADS) supplement for CEM was published in 2022 [12]; this supplement, in part, outlines a lexicon to describe findings on CEM, with descriptors adapted from the mammography and MRI lexicons for findings identified on low-energy images and recombined images, respectively.

Lesion conspicuity is defined as the degree of enhancement relative to background parenchymal enhancement (BPE), and is subjectively graded on CEM recombined images as low, moderate, or high, according to the ACR BI-RADS supplement for CEM [12, 13]. A low-conspicuity lesion enhances equal to or slightly greater than BPE, while a high-conspicuity lesion enhances much greater than the background. A moderate-conspicuity lesion falls between low and high conspicuity.

The criteria for predicting benignity vs malignancy of lesions identified on CEM are still being defined. The purpose of this study was to determine if lesion conspicuity on CEM is independently associated with malignancy.

## Materials and methods

### Study design and sample

This single-center, retrospective study was approved by the institutional review board and was compliant with the United States Health Insurance Portability and Accountability Act. The institutional database was queried for consecutive diagnostic and screening CEM examinations performed between January 2019 and December 2021, resulting in 4553 CEM examinations performed in 3134 patients. The inclusion criterion was a CEM examination with a BI-RADS assessment of 0, 3, 4, or 5 (*n* = 757) at the time of initial radiologist interpretation. From 757 CEM examinations, 281 were excluded for the following reasons: CEM examination performed in a patient with biopsy-proven malignancy for evaluating the extent of disease or performed for short-term follow-up of a previously identified finding; or CEM examination lacking two-year imaging follow-up and/or biopsy pathology results. In total, 476 CEM examinations containing 563 enhancing lesions in 455 patients were included in the study sample. Overlap in the study sample with previous studies is described in the Electronic Supplementary Material.

### CEM examinations

CEM examinations were performed using FDA-approved mammography systems with CEM capability, including 3Dimensions and Selenia Dimensions (Hologic), and Senographe Pristina and Senographe Essential (GE Healthcare). Prior to imaging acquisition, iodinated contrast (iohexol, 350 mg I/mL) was injected intravenously at a rate of 3 mL/s. Imaging began 2.5 min after injection, with near-simultaneous acquisition of low- and high-energy images for each view. Mediolateral oblique and craniocaudal views of both breasts were obtained within 8 min after injection, or of the remaining breast in patients with a history of unilateral mastectomy. For each view, both a low-energy image (equivalent to a full-field digital mammogram) and a generated recombined image (demonstrating contrast enhancement) were made available for interpretation. In accordance with the ACR BI-RADS manual [13], for a bilateral breast examination, the same view is alternated between the breasts so that both breasts are imaged when contrast is maximally present. In general, at our institution, the right craniocaudal projection is obtained first, followed by the left craniocaudal, and then the right and left mediolateral oblique projections.

### Retrospective review of CEM examinations

The 476 CEM examinations were divided between two breast radiologists (T.A. and C.H.L., with 7 and 13 years of experience of interpreting CEM) who performed independent retrospective review, blinded to two-year imaging follow-up or biopsy pathology results. For each examination, the assigned radiologist graded the conspicuity (low, moderate, high) of each enhancing lesion previously identified by the original interpreting breast radiologist on the recombined image, as well as recorded the enhancement pattern (asymmetry, mass, non mass enhancement with or without calcifications) and if the enhancing lesion was present on the recombined image only or also had a low-energy correlate (i.e., the lesion was present on both the recombined and low-energy images), according to the ACR BI-RADS supplement for CEM [12]. Date of birth, breast cancer risk factors, mammographic breast density, and BPE assigned by the initial interpreting radiologist were collected from the electronic medical record. The CEM vendor was recorded from the DICOM header. Subsequently, 100 randomly selected lesions were reviewed independently by both radiologists to determine inter-reader agreement. Lesions with discrepant gradings were re-reviewed in consensus to reach a consensus assessment.

### Statistical analysis

Descriptive statistics were used to summarize patient demographics and clinical characteristics, using medians and IQRs for continuous variables, and counts and percentages for categorical variables. The positive predictive value (PPV) for malignancy was determined for each level of conspicuity (low, moderate, high), along with 95% CIs. The PPV was estimated as the proportion of cancers within each category of conspicuity. *P-*values to assess the difference in PPV were estimated using a generalized estimating equations (GEE) framework with an exchangeable correlation matrix, to account for intraindividual correlation. The model consisted of cancer diagnosis (0 = benign; 1 = malignant) as the outcome variable and level of conspicuity as the explanatory variable. This comparison was made using a pairwise approach (low vs moderate, moderate vs high, and high vs low). Similarly, lesion-level features are compared between the three levels of conspicuity using an ordinal GEE model, with ordered conspicuity levels as the outcome variable and the corresponding feature as the explanatory variable. This analysis was conducted using the *geepack* package in R 4.5 [14]. In addition, to evaluate for confounders, a multivariable logistic regression analysis was conducted with diagnosis of malignancy as the outcome variable and modality, conspicuity, risk factor, BPE, and breast density as the explanatory variables, in a GEE framework. Inter-reader agreement was assessed using Cohen’s kappa among the 100 lesions that were evaluated by both readers. All presented statistical analysis was conducted using R 4.5 software. The type I error rate (α) was set to 0.05.

## Results

### Study sample characteristics

**Fig. 1:**
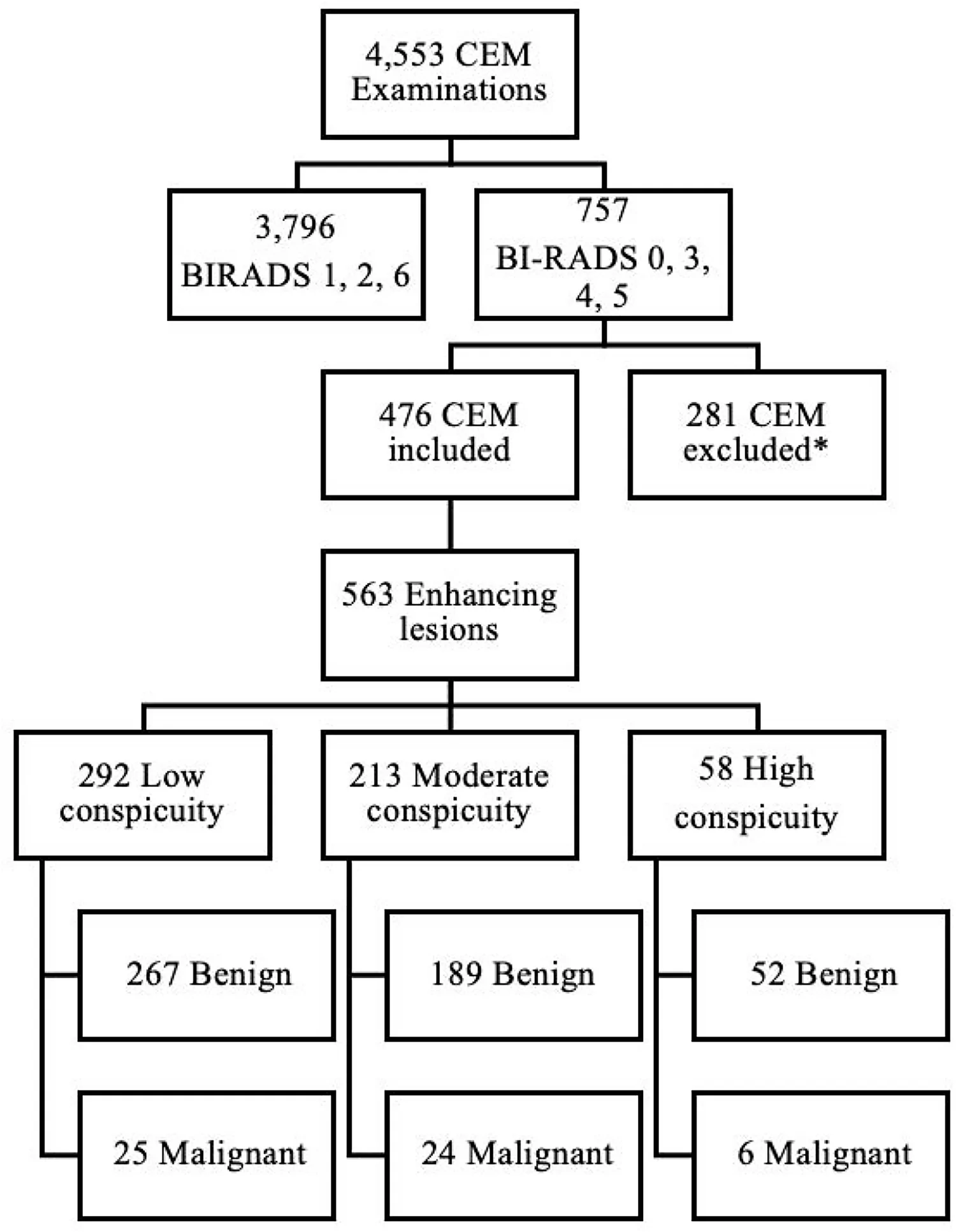
Study Inclusion. 281 CEM examinations were excluded for the following reasons: newly diagnosed breast cancer, follow-up for a previously evaluated lesion on CEM, or lacking two-year imaging follow-up or biopsy pathology results. CEM, contrast-enhanced mammography

The final study sample comprised 455 women (median age, 49 years [IQR: 44–57]). Based on two-year imaging follow-up or biopsy pathology results, among 563 enhancing lesions in 476 CEM examinations, 55 were malignant, and the remaining 508 were benign. Figure 1 shows the flow of patient inclusion into the study. Most examinations were performed in women with dense breasts (91%, 433/476), with 42% (199/476) demonstrating moderate or marked BPE. Regarding breast cancer risk factors, 54% of patients had a personal history of breast cancer (245/455), while 23% had a personal history of a high-risk lesion (106/455). 94% (448/476) of CEM examinations were routine screening examinations performed in asymptomatic women. Slightly more examinations were performed on a GE unit (67%, 324/482) than on a Hologic unit (33%, 148/482). Table 1 summarizes the study sample characteristics.

**Table 1:** Patient- and examination-Level characteristics

Characteristic	*n* (%)
Patient demographics (*n* = 455)	
Female	455 (100)
Age (y)	49 (44–57)^*^
Risk factors	
Personal history	245 (53.8)
Family history	82 (18.7)
High-risk lesion	106 (23.3)
Genetic mutation	9 (2.0)
Dense breasts	11 (2.4)
Unknown	2 (0.4)
Exam characteristics (*n* = 476)	
Clinical indication for CEM	
Elevated risk screening	448 (94.1)
Diagnostic examination	28 (5.9)
Palpable lump	16 (57.1)
Pain	3 (10.7)
Nipple discharge	2 (7.2)
Other^†^	7 (25.0)
Breast density	
Non-dense	43 (9.0)
Dense	433 (91.0)
BPE	
Minimal/mild	277 (58.2)
Moderate/marked	199 (41.8)

Except where indicated, data are raw numbers, with percentages in parentheses

*BPE* background parenchymal enhancement, *CEM* contrast-enhanced mammography

^*^ Data are medians, with IQR in parentheses

^†^ Patients were scheduled for diagnostic CEM for follow-up of a finding separate/distinct from the lesion evaluated for this study (e.g., follow-up for calcifications in the contralateral breast)

### Lesion conspicuity

Table 2 summarizes the results regarding lesion conspicuity and malignancy across all 563 enhancing lesions. Lesions were most frequently graded as having low conspicuity (52%, 292/563), followed by moderate conspicuity (38%, 213/563), and high conspicuity (10.3%, 58/563). Whether low, moderate, or high conspicuity, the PPV to predict malignancy was low: 9% (95% CI: 6–12%), 11% (95% CI: 7–16%), and 10% (95% CI: 4–21%), respectively. Across all lesions, there was no significant difference in the PPV between low and high conspicuity (*p* = 0.68), low and moderate conspicuity (*p* = 0.34), or moderate and high conspicuity (*p* = 0.83). Further, the level of conspicuity did not differ significantly between benign and malignant lesions in various lesion subgroups, including lesions presenting on recombined images only (*p* = 0.13), lesions presenting on both recombined and low-energy images (*p* = 0.07), lesions presenting as enhancing asymmetry (*p* = 0.9), lesions presenting as mass enhancement (*p* = 0.5), or lesions presenting as non-mass enhancement (*p* = 0.6). When evaluating risk factors, breast density, and BPE as potential confounders, the multivariable model adjusting for these factors was not significant. The results are provided in Supplemental Table S1.

**Table 2:** Lesion conspicuity on CEM and benign vs malignant outcomes

	Conspicuity			*p*-value
	Low	Moderate	High	
Pathology or 2-year imaging follow-up results				0.34–0.83^‡^
Benign	267 (91)	189 (89)	52 (90)	0.65^**^
Malignant	25 (9)	24 (11)	6 (10)	
PPV for malignancy^*^	9 (6, 12) 25/292	11 (7, 16) 24/213	10 (4, 21) 6/58	
Presence on RI and LE images				
RI only				0.13
Benign	229 (91.6)	163 (90.6)	38 (97.4)	
Malignant	21 (8.4)	17 (9.4)	1 (2.6)	
RI + LE images				0.07
Benign	38 (90.5)	26 (78.8)	14 (73.7)	
Malignant	4 (9.5)	7 (21.2)	5 (26.3)	
Enhancement pattern^†^				
Asymmetry				n/a
Benign	94 (92.2)	47 (94.0)	12 (100)	
Malignant	8 (7.8)	3 (6.0)	0 (0)	
Mass				0.73
Benign	50 (89.3)	59 (81.9)	20 (87)	
Malignant	6 (10.7)	13 (18.1)	3 (13)	
Focal NME				n/a
Benign	121 (93)	82 (92.1)	20 (100)	
Malignant	9 (7)	7 (7.9)	0 (0)	

Except where indicated, data are raw numbers, with percentages in parentheses

n/a indicates that *p*-values were not calculated due to low cell counts

Total *n* lesions = 563

*LE* low-energy, *RI* recombined

^*^ Data are percentages, with 95% confidence intervals in parentheses

^**^ *p*-values are calculated using a GEE model

^†^ Data reflect non-calcified lesions only

^‡^ Calculated to compare PPV values: low vs moderate conspicuity, *p* = 0.34; low vs high conspicuity, *p* = 0.68, and moderate vs high conspicuity, *p* = 0.83. *p*-values are calculated using a GEE model

Table 3 summarizes the results regarding lesion conspicuity across only the 55 malignant enhancing lesions. Among these lesions, 14 (25.5%) were ductal carcinomas in situ and 41 (74.5%) were invasive cancers (including 4 microinvasive carcinomas); there was no significant difference in the level of conspicuity between ductal carcinomas in situ and invasive carcinomas (*p* = 0.92). There was also no significant difference in conspicuity based on cancer enhancement pattern (*p* = 0.79). However, most high-conspicuity cancers presented on both recombined and low-energy images (5/6; 83%), while most low- (21/25; 84%) and moderate-conspicuity cancers (17/24; 71%) presented on recombined images only (*p* = 0.006).

**Table 3:** Cancer lesion conspicuity and characteristics on CEM

	Conspicuity			*p*-value
	Low	Moderate	High	
Pathology				0.92
DCIS	9 (36.0)	3 (12.5)	2 (33.3)	
Invasive	16 (64.0)	21 (87.5)	4 (66.7)	
Presence on RI and LE images				
RI only	21 (84)	17 (71)	1 (17)	0.006
RI + LE images	4 (16)	7 (29)	5 (83)	
Cancer enhancement pattern				
Asymmetry	8 (32)	3 (12.5)	0 (0)	0.79^*^
Mass	6 (24)	13 (54.2)	3 (50)	
NME without calcifications	9 (36)	7 (29.2)	0 (0)	
NME with calcifications	2 (8)	1 (4.2)	3 (50)	

*n* = 55; data are raw numbers, with percentages in parentheses

*DCIS* ductal carcinoma in situ, *LE* low-energy, *NME* non-mass enhancement, *RI* recombined

*p*-values are calculated using a GEE model

* This *p*-value is estimated after combining categories of asymmetry and mass into one and NME into the other. This is done because zero counts in certain categories make the *p* values non-estimable

**Fig. 2:**
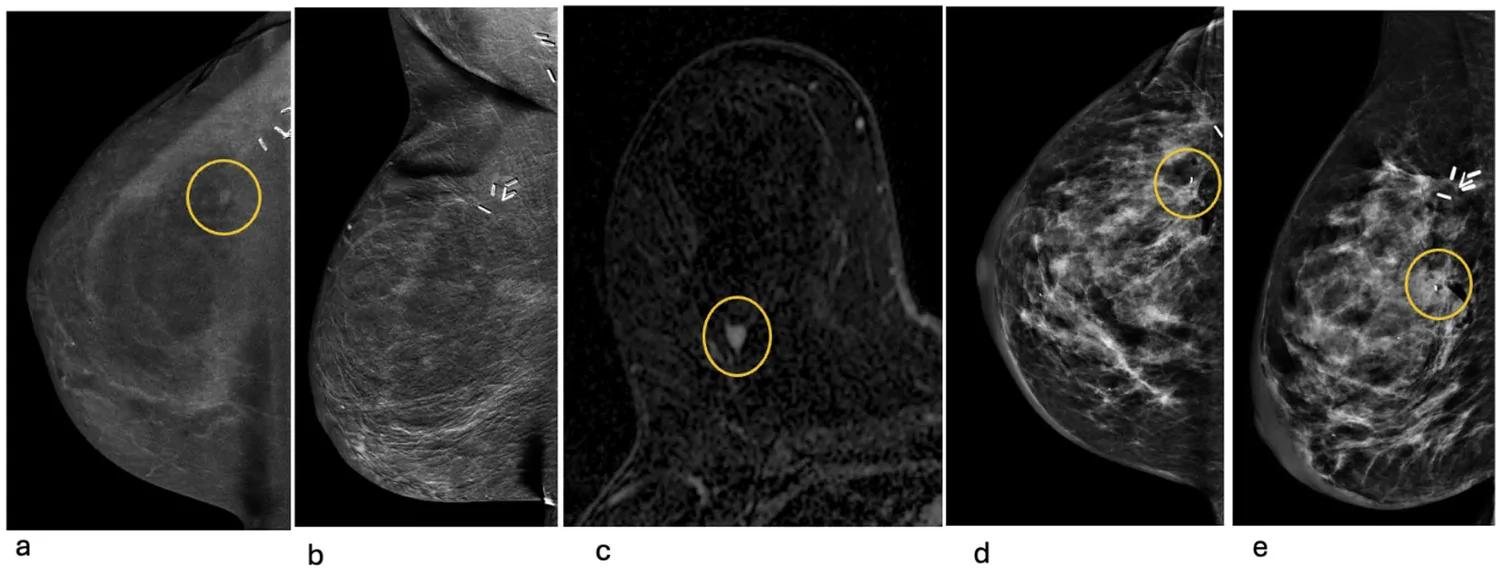
Low-conspicuity enhancing invasive lobular carcinoma. A 63-year-old woman with a history of right breast cancer and lumpectomy underwent routine screening CEM. On CEM, a low-conspicuity enhancing asymmetry is seen on the recombined images on the craniocaudal projection (**a**), without a clear, definite correlate on the mediolateral projection (**b**) or on the low-energy images (not pictured). Subsequent contrast-enhanced MRI (**c** axial post-contrast subtraction sequence) demonstrated a corresponding enhancing mass. MRI-guided biopsy was performed, yielding invasive lobular carcinoma. Post-procedure full-field digital mammogram (**d** craniocaudal projection and **e** lateral projection) demonstrated the biopsy marker in the location of the originally identified enhancing asymmetry

**Fig. 3:**
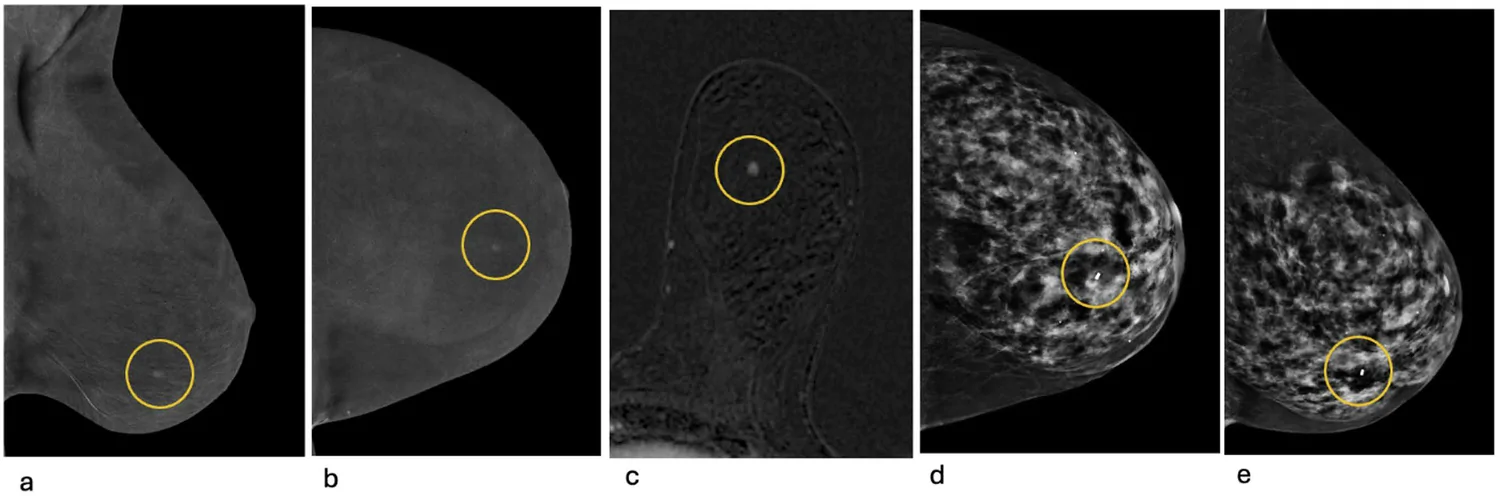
Low-conspicuity enhancing invasive carcinoma (not otherwise specified). A 76-year-old woman with a family history of breast cancer underwent routine screening CEM. On CEM, a low-conspicuity enhancing mass is seen on the recombined images, on both mediolateral oblique (**a**) and craniocaudal (**b**) projections, without a correlate on the low-energy images (not pictured). Subsequent contrast-enhanced MRI (**c** axial post-contrast subtraction sequence) demonstrated a corresponding enhancing mass. MRI-guided biopsy was performed, yielding invasive ductal carcinoma. Post-procedure full-field digital mammogram (**d** craniocaudal projection and **e** lateral projection) demonstrated the biopsy marker in the location of the originally identified enhancing mass

**Fig. 4:**
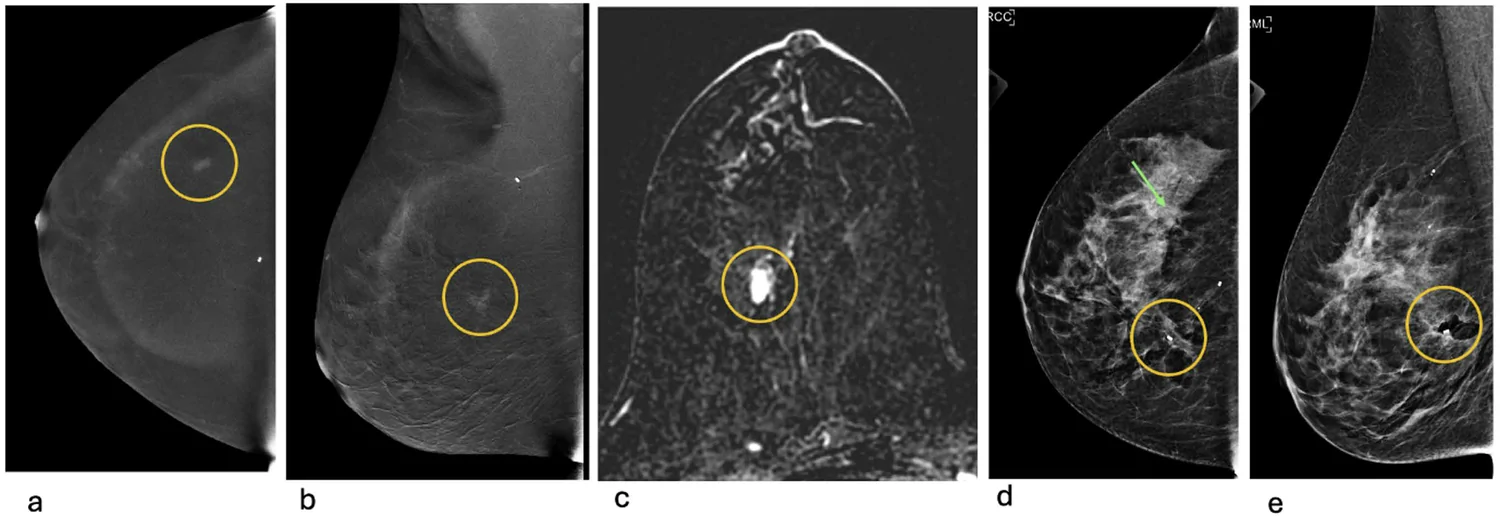
Moderate-conspicuity invasive carcinoma (not otherwise specified). A 58-year-old woman with a family history of breast cancer underwent routine screening CEM. On CEM, a moderate-conspicuity focal non-mass enhancement is seen on recombined images, on the craniocaudal (**a**) and mediolateral oblique (**b**) projections, without a correlate on the low-energy images (not pictured). Subsequent contrast-enhanced MRI (**c** axial post-contrast subtraction sequence) demonstrated a corresponding enhancing mass. MRI-guided biopsy was performed, yielding invasive carcinoma (not otherwise specified). Post-procedure full-field digital mammogram (**d** craniocaudal projection and **e** lateral projection) demonstrated the biopsy marker in the location of the originally identified enhancing mass

**Fig. 5:**
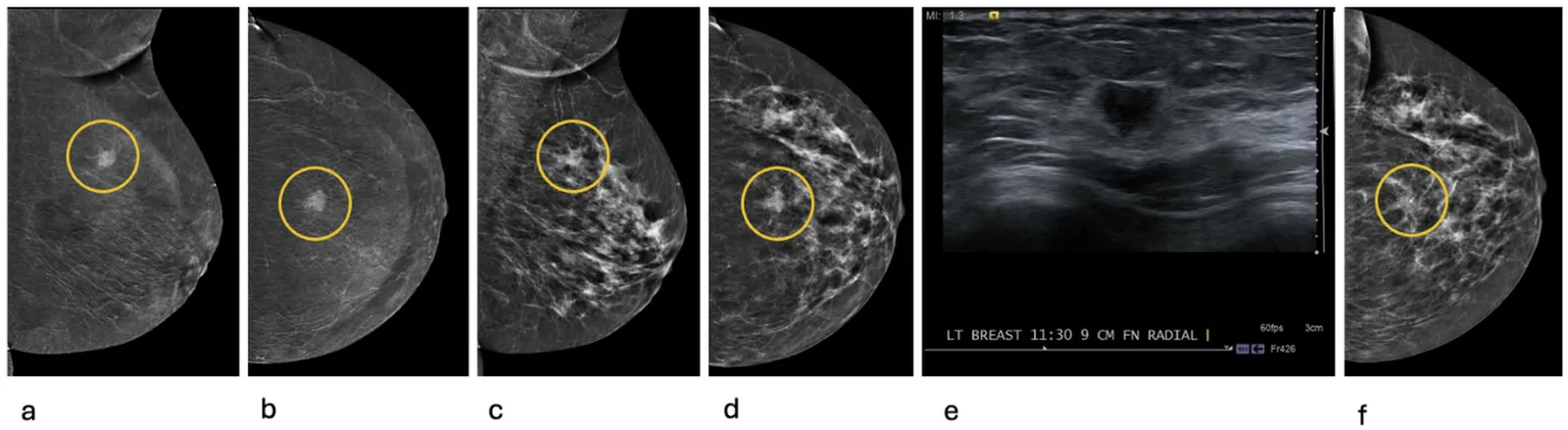
High-conspicuity invasive carcinoma (not otherwise specified). A 70-year-old woman with a history of left breast excision for pleomorphic lobular carcinoma in situ and atypical ductal hyperplasia underwent routine screening CEM. On CEM, a high-conspicuity enhancing mass is seen on the recombined images, on both mediolateral oblique (**a**) and craniocaudal (**b**) projections, with a low-energy correlate on both mediolateral oblique (**c**) and craniocaudal (**d**) projections. Diagnostic ultrasound (**e**) demonstrated a suspicious mass, which underwent ultrasound-guided core biopsy, yielding invasive carcinoma (not otherwise specified). Post-procedure full-field digital mammogram (**f** craniocaudal projection) demonstrated the biopsy marker in the location of the originally identified enhancing mass

**Fig. 6:**
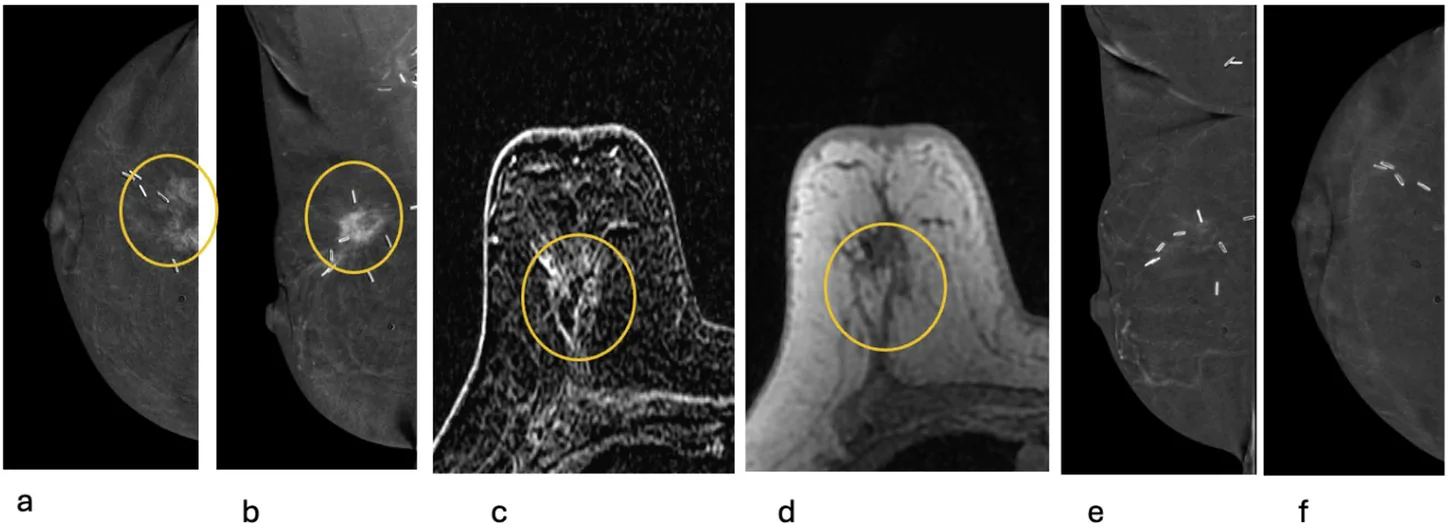
High-conspicuity fat necrosis. A 60-year-old woman with a history of right breast cancer status and lumpectomy underwent routine screening CEM. On CEM, a high-conspicuity peripherally enhancing mass is seen in the lumpectomy bed on the recombined images, on both craniocaudal (**a**) and mediolateral (**b**) projections. Subsequent contrast-enhanced MRI (**c** axial post-contrast subtraction sequence, and **d** T1-weighted non-contrast sequence) demonstrated peripheral enhancement with central fat, consistent with benign fat necrosis. Follow-up CEM at two years (**e** craniocaudal projection and **f** mediolateral oblique projection) demonstrated resolving enhancement in the lumpectomy bed, further supporting a benign etiology

Figures 2–4 depict low- and moderate-conspicuity cancers, Fig. 5 depicts a high-conspicuity cancer, and Fig. 6 a high-conspicuity benign lesion.

### Inter-reader agreement

Of 100 lesions whose conspicuity was graded by both breast radiologists, there was 93% (93/100) agreement. The κ value for conspicuity agreement was 0.94 (*p* = 0.001), indicating almost-perfect agreement.

## Discussion

Our results demonstrate that lesion conspicuity as seen on CEM recombined images is not independently associated with benignity or malignancy, with a PPV to predict malignancy of 8.6% (95% CI: 5.6–12.4%), 11.3% (95% CI: 7.4–16.3%), and 10.3% (95% CI: 3.9–21.1%) for low, moderate, and high conspicuity, respectively. Differences in PPV between low, moderate, and high conspicuity were not significant (low vs high conspicuity*, p* = 0.68; low vs moderate conspicuity, *p* = 0.34; moderate vs high conspicuity, *p* = 0.83). Most enhancing lesions on CEM recombined images, including most cancers, showed low or moderate conspicuity. Compared with high-conspicuity lesions, those with low or moderate conspicuity most often presented on recombined images alone, without a correlate on the low-energy images.

Our results conflict with multiple prior studies reporting that higher lesion conspicuity on CEM is correlated with a higher likelihood of malignancy [15–19]. This may be partially due to differences in study design and sample. Both Dharmalingam et al and Deng et al’s studies included patients with known lesions initially identified on digital breast tomosynthesis or ultrasound prior to CEM. In our study, 83% (469/563) of the enhancing lesions were present on recombined images only, without a correlate on the low-energy images. In our study, cancers with a low-energy correlate (similar to the samples included in the Dharmalinga et al and Deng et al studies) tended to have a high conspicuity level of enhancement. Rudniki et al [19] included patients with either mammogram or ultrasound findings suspicious for multicentric/multifocal cancer. Such patients were excluded from our study. Bellini et al [20] included 552 biopsy-proven cancers and found significant associations between cancer lesion conspicuity and enhancement type, particularly that mass-forming cancers were more conspicuous, while non-mass enhancement resulted in lower conspicuity. In contrast, our study included all abnormal findings with enhancement, both cancers and enhancement due to benign etiologies. Additionally, in our study, lesions were evaluated on CEM prior to biopsy, while in Bellini et al’s study, CEM was performed after biopsy, which may alter the vascular environment of the tumor. For instance, post-biopsy changes may increase tumor conspicuity, particularly on a 2D view with summation of overlapping enhancement, or the biopsy procedure may alter the vascular permeability of the tumor and the immediate surrounding parenchyma.

Unlike MRI, in which both breasts can be imaged simultaneously in the axial plane, the order of image acquisition in CEM varies between sides and planes. This makes lesion conspicuity subject to the unique enhancement kinetics of the lesion at the time of image acquisition. Additionally, it should be noted that enhancement on CEM is not an absolute iodine quantification. Two images are acquired after the administration of iodinated intravenous contrast: one high-energy image with average x-ray energy above the 33.2 keV K-edge of iodine, and one low-energy image acquired with x-ray energies consistent with typical full-field digital mammograms below the K-edge of iodine. An algorithm involving the logarithmic transformation and weighted subtraction of the high-energy and low-energy images is then employed to generate the recombined image displaying areas of contrast enhancement. The performance of this algorithm varies based on different characteristics of the breast, such as thickness and composition, and varies across vendors [21, 22]. Thickness and density vary across the breast tissue; thus, the accuracy of these algorithms will also affect the level of lesion conspicuity. Further, a dense, thick breast classically produces more scatter with a reduced signal-to-noise ratio [23], which could affect the subtraction algorithm and background cancellation [24]. Misregistration, scatter, and high attenuation artifacts are all known imperfections of the technical and software processing algorithms of CEM [25].

Our institution uses both GE and Hologic units, with approximately two-thirds of the examinations performed on GE. Recent studies comparing CEM manufacturers have found that GE systems exhibit a linear response (coefficient of determination *R*^2^ ≥ 0.98) between image pixel values and iodine concentration, while Hologic systems display a weaker linear response (*R*^2^ ≤ 0.97) that may be better characterized as a quadratic response [24, 26, 27]. Additionally, beyond the differences in hardware between vendors, there are post-processing considerations, with differences based on the CEM software version employed at the time for each unit. While the technical post-processing details are unique to each vendor, largely proprietary, and beyond the scope of this paper, the clinically meaningful result is that lesion conspicuity is likely not solely due to lesion vascularity and/or tumor angiogenesis. Therefore, low-conspicuity lesions should not be presumed to be benign based on conspicuity alone.

Our study has a few limitations. First, this is a single-institution study performed at a dedicated cancer center with dedicated breast radiologists. Over 50% of the study sample had a personal history of breast cancer, over 20% had a history of a high-risk lesion, and nearly 20% had a family history of breast cancer. Our results are exploratory and may not be generalizable to a community setting or another academic setting with fewer post-operative patients, or a patient population with a different risk profile. Second, this was a retrospective reader study reflecting the CEM protocol at our institution, predominantly for routine screening. There was no “breast of interest” to be imaged first. Third, the CEM examinations were obtained using two different vendors and four CEM equipment models. Overall, larger studies are needed, with varied patient populations represented, to continue testing the results of our study.

In conclusion, lesion conspicuity alone, as determined on CEM recombined images, is not associated with benignity or malignancy. Most of the enhancing lesions on CEM recombined images, including most cancers, demonstrated low or moderate conspicuity. Any enhancing lesion on a CEM recombined image should not be discounted based on low conspicuity alone, even if lacking a correlate on the low-energy images, as most low-conspicuity cancers presented with recombined image enhancement only.

## Supplementary information

ELECTRONIC SUPPLEMENTARY MATERIAL

## Abbreviations

BI-RADS: Breast imaging reporting and data system
BPE: Background parenchymal enhancement
CEM: Contrast-enhanced mammography
FDA: Food and Drug Administration
GEE: Generalized estimating equations
PPV: Positive predictive value
*R*^2^: Coefficient of determination
