# Supplementary material for: Clinical significance of lesion conspicuity on contrast-enhanced mammography
Source: Eur Radiol. 2026 Jun 13;36(10):7864–73. doi: 10.1007/s00330-026-12660-y (PMC13574885; doi:10.1007/s00330-026-12660-y)
Supplement: Supplementary file 1 [file 330_2026_12660_MOESM1_ESM.docx]

**SUPPLEMENTAL CONTENT**

**Supplemental Methods**

***Patient Overlap***

Patients in our study overlapped with prior retrospective studies as follows: 448 patients overlapped with Amir et al.’s study in on interval cancers after negative CEM [1], 155 patients overlapped with Nissan et al.’s study on the sensitivity and specificity of CEM in extremely dense breasts [2], 71 patients overlapped with Coffey et al.’s study [3] on the utility of short-term follow-up of CEM lesions after a negative MRI, and 10 patients overlapped with Hogan et al.’s study on the role of CEM in patients with a history of lobular neoplasia [4]. In addition, 9 patients overlapped with Horvat et al.’s prospective study [5] on the role of supplemental whole-breast ultrasound with CEM.

References

1.	Amir T, Lee CH, Eskreis-Winkler S, et al. Interval Cancers after Negative Screening Contrast-enhanced Mammography. *Radiol Imaging Cancer* 2026; 8:e250559

2.	Nissan N, Comstock CE, Sevilimedu V, et al. Diagnostic Accuracy of Screening Contrast-enhanced Mammography for Women with Extremely Dense Breasts at Increased Risk of Breast Cancer. *Radiology* 2024; 313:e232580

3.	Coffey K, Dixon LB, Sevilimedu V, Jochelson MS, Sung JS. Short-term follow-up of contrast-enhanced mammography lesions after negative breast MRI in women with elevated breast cancer risk. *Eur J Radiol* 2023; 168:111097

4.	Hogan MP, Amir T, Sevilimedu V, Sung J, Morris EA, Jochelson MS. Contrast-Enhanced Digital Mammography Screening for Intermediate-Risk Women With a History of Lobular Neoplasia. *AJR Am J Roentgenol* 2021; 216:1486–1491

5.	Horvat JV, Amir T, Watt GP, et al. Comparison of Contrast-enhanced Mammography and Low-Energy Imaging with or without Supplemental Whole-Breast US in Breast Cancer Detection. *Radiology* 2025; 314:e242006

**Supplemental Table S1. Multivariable analysis evaluating risk factors, breast density, and background parenchymal enhancement as potential confounders**

| Characteristic | OR (95% CI) | *P*-value |
| --- | --- | --- |
| Conspicuity |  |  |
| Low | - | 0.70 |
| Moderate | 1.34 (0.71, 2.51) |  |
| High | 1.0 (0.34, 2.63) |  |
| Risk factor |  | 0.60 |
| High-risk lesion | - |  |
| Personal history | 1.05 (0.50, 2.21) |  |
| Family history | 0.64 (0.22, 1.85) |  |
| Dense breasts/unknown | 0.42 (0.04, 4.29) |  |
| Breast Density |  | 0.10 |
| Dense | - |  |
| Not dense | 2.03 (0.88, 4.72) |  |
| BPE |  | 0.15 |
| Minimal/mild | - |  |
| Moderate/marked | 0.59 (0.29, 1.20) |  |

Note.—BPE = background parenchymal enhancement, CI = confidence interval, OR = odds ratio
